# Role of Polyphenols and Other Phytochemicals on Molecular Signaling

**DOI:** 10.1155/2015/504253

**Published:** 2015-06-09

**Authors:** Swapna Upadhyay, Madhulika Dixit

**Affiliations:** Laboratory of Vascular Biology, Department of Biotechnology, Bhupat and Jyoti Mehta School of Biosciences and Bioengineering Building, Indian Institute of Technology, Madras, Chennai, Tamil Nadu 600036, India

## Abstract

Optimized nutrition through supplementation of diet with plant derived phytochemicals has attracted significant attention to prevent the onset of many chronic diseases including cardiovascular impairments, cancer, and metabolic disorder. These phytonutrients alone or in combination with others are believed to impart beneficial effects and play pivotal role in metabolic abnormalities such as dyslipidemia, insulin resistance, hypertension, glucose intolerance, systemic inflammation, and oxidative stress. Epidemiological and preclinical studies demonstrated that fruits, vegetables, and beverages rich in carotenoids, isoflavones, phytoestrogens, and phytosterols delay the onset of atherosclerosis or act as a chemoprotective agent by interacting with the underlying pathomechanisms. Phytochemicals exert their beneficial effects either by reducing the circulating levels of cholesterol or by inhibiting lipid oxidation, while others exhibit anti-inflammatory and antiplatelet activities. Additionally, they reduce neointimal thickening by inhibiting proliferation of smooth muscle cells and also improve endothelium dependent vasorelaxation by modulating bioavailability of nitric-oxide and voltage-gated ion channels. However, detailed and profound knowledge on specific molecular targets of each phytochemical is very important to ensure safe use of these active compounds as a therapeutic agent. Thus, this paper reviews the active antioxidative, antiproliferative, anti-inflammatory, or antiangiogenesis role of various phytochemicals for prevention of chronic diseases.

## 1. Introduction

Overwhelming evidence from epidemiological,* in vivo, in vitro*, and clinical trial data indicates that the plant-based diet can reduce the risk of chronic diseases (e.g., cardiovascular disease, hypertension, diabetes, and cancer) due to presence of biologically active plant compounds or phytochemicals. Steinmetz and Potter identified more than a dozen classes of phytochemicals from plant based diets (fruits, vegetable, nuts, etc.), such as carotenoids, phenolic compounds (flavonoids, isoflavonoids, and lignin), phenolic acid, phytosterols and phytostanols, tocotrienols, organosulfur compounds, and nondigestible carbohydrates (dietary fiber, Table 1) [1]. According to many researchers regular consumption of diet rich in vegetables, fruits, whole grains, herbs, nuts, seeds, which contain plenty of phenolic compounds, terpenoids, phytosterols, organosulfur exert their beneficial effect in disease prevention, by regulating several cellular molecular pathways like regulation of inflammation, redox potentials, metabolic disorder, apoptosis, and so forth [2]. Polyphenols (flavonoids, lignans, stilbenes, etc., see Table 1) are the most diverse group of phytochemicals distributed in vegetables, fruits, olive oil, and wine and exhibit wide range of protective roles such as hypolipidemic, antioxidative, antiproliferative, and anti-inflammatory effects to reduce the onset of disease progression [3–5]. Similarly terpenoids (carotene, lycopene, etc.), another important chemically active natural hydrocarbon, contain oxygen rich moieties like alcohol, aldehydes, and ketones. Epidemiological studies have shown that the oral supplementation of tomato extract (rich in carotenoid, lycopene) significantly control the risk of hyperlipidemia, CVD, metabolic syndrome by regulating several physiological phenonmenon like reduction of blood pressure of low density lipoprotein oxidation, hypertension [6]. Organosulfur, phytosterols are the other widely distributed active phytochemicals which have been found to reduce the generation of inflammatory signaling molecules and also modulate antioxidative effect by inhibition of NF-*κ*B pathways [6, 7]. These phytochemicals (organosulfur, phytosterols) also show protective effect against atherosclerosis by regulating of serum total and LDL-cholesterol levels. Phytochemicals such as resveratrol, epigallocatechin gallate, gingerol, phytosterol, and myricetin directly influence various molecular signal transduction pathways like inflammation cascade, cell proliferation/migration, oxidative stress, and metabolic disorders, which are involved in the development of several noncommunicable diseases. Although beneficial effects of plant based diets or phytochemicals in reducing the risk of chronic diseases have been shown through various epidemiological and* in vitro*/*in vivo* studies, much more mechanistic and clinical evidence is required to define a particular phytochemical as an inhibitor of specific cellular pathways or identify the plant derived active compound with specific therapeutics properties. Therefore, this review is focused on the cellular targets of naturally occurring active compounds from various sources in order to explore the protective/therapeutics role as an antioxidative, anti-inflammatory regulation of metabolic disorder as well as antiproliferative properties by targeting specific molecular signal transduction, which might play a crucial role in the pathogenesis of chronic disease.

## 2. Bioavailability of Phytochemicals

Bioavailability of individual compounds of interest at the target site is one of the important challenges/parameters to determine the therapeutic efficiency of the target drug [8]. Although accurate bioavailability of particular compound is not possible to predict according to Lipinski et al. a compound might have better bioavailability when it contains maximum 5 hydrogen-bond donors, and 10 hydrogen-bond acceptors, in association with having a molecular mass not more than 500 daltons; a partition coefficient log *P* value should not be more than 5 and contains less than 10 rotatable bonds [9]. Most of the phytochemicals, including polyphenols such as curcumin and green tea polyphenols, do not satisfy all these chemical specifications and exhibit low bioavailability [10]. Although compounds such as genistein and biochanin A have all those good absorptive chemical properties, high rate excretion in the gut by efflux mechanism limits their bioavailability [10]. Therefore, other factors like solubility of the compounds, stability due to gastric and colonic pH, metabolism by gut microflora, absorption across the intestinal wall, active efflux mechanism, and first-pass metabolic effects may also play crucial role in limiting the bioavailability of phytochemicals [11]. As an example, limited bioavailability of epigallocatechin gallate is mainly due to poor absorption and rapid first-pass metabolism. Similarly low stability, increased oxidation, and high hepatic uptake are also responsible for the limited the bioavailability of some widely distributed polyphenols such as flavonoids (flavonols, anthocyanines) resveratol 8 (grapes) [12].

Moreover it has been also reported that rapid conjugation of a phytochemicals, especially by glucuronidation in the intestine and liver, in association with reaction of cytochrome P450 (C-P450) enzymes, which are recognized as important clearance mechanisms, is primarily responsible for their poor bioavailability [12]. Many researchers have shown that codelivery of lead target molecules (phytochemicals) with an agent that can modulate the activity of glucuronidation or inhibit C-P450 mediated clearance mechanism is possible to increase the bioavailability of active compound of interest at the target site [12, 13]. Piperine, a component of black pepper, inhibits glucuronidation and is able to enhance the bioavailability of several bioactive compounds by altering the C-p450 mediated enzymatic biotransformation [8, 14]. Rapid mobilization in the intestine is largely responsible for low bioavailability of curcumin at target site; however consumption of curcumin along with piperin, a known inhibitor of intestinal and hepatic *β*-glucuronidation of curcumin, may induce the bioavailability of curcumin about 20-fold [8, 14]. Many phytochemicals were identified as promising therapeutics agent in the preliminary* in vitro* studies. However, when those compounds were tested into* in vivo* studies, many of them failed to translate the preclinical/clinical findings, because compounds either were unstable in the gut or exhibited poor bioavailability [8, 14]. Therefore, detailed preclinical and clinical studies on bioavailability of phytochemicals/active compounds are urgently needed to understand their therapeutic limitation as well as to find out the better compound delivery system to achieve the best efficiency level of the drug at the target organ.

## 3. Molecular Mechanism of Photochemical in Redox Modulation

Cells use enzymes and oxygen to perform its normal physiological functions are continuously exposed to free radicals. Free radicals are oxygen containing highly reactive molecules with one or more unpaired electrons. This highly reactive oxygen species (ROS) includes numerous partially reduced oxygen metabolites such as hydrogen peroxide (H_2_O_2_), hydroxyl radicals (*∙*OH), and superoxide anions (O∙^−^_2_) radicals. ROS are generated intracellularly as a byproduct of normal metabolism and as second messengers in various signal transduction pathways [15]. ROS are also generated exogenously due to direct uptake by cells from the extracellular sources or produced during exposure of cells to some environmental triggers. It has been well established that ROS are heterogeneous and can exert their beneficial or detrimental effect depending on the concentrations at which they are present in the cellular level. At low levels, ROS can be driven by NADPH/NADPH oxidase and is required to maintain homeostatic signaling events as well as inducing cell proliferation and survival through the posttranslational modification of kinases and phosphatase [16]. However, overproduction of ROS or exposure of cells to ROS for extended period may cause irreversible damage to DNA, protein, and lipids. Therefore, numerous innate defense systems have developed to detoxify or prevent the detrimental effect of ROS. These include nonenzymatic molecules (e.g., glutathione, vitamins A, C, and E, and flavonoids) as well as induction of phase II detoxifying/antioxidative enzymes (e.g., superoxide dismutase (SOD), catalase, and glutathione peroxidase (GSH-Px) and hemeoxygenase-1 (HO-1), which are involved to eliminate or inactivate the ROS from cellular level) [17]. An imbalance between ROS generation and defense mechanism or inadequate presence of antioxidant molecules results in the state known as oxidative stress. Growing evidence indicates that chronic and acute excess generation of ROS under pathophysiologic conditions is pivotal in the development of cardiovascular diseases (CVD) or premature atherosclerosis progression, cancer, pulmonary fibrosis, and neurodegenerative disorder [18–20]. Oxidative modification of low density lipoprotein (Ox-LDL) regulates many signaling pathways, which causes inhibition of endothelial nitric oxide synthase (eNOS) and promotes vasoconstriction, expression of adhesion molecules, and progression of platelet aggregation. Additionally proliferation of smooth muscle cell (SMC) by Ox-LDL stimulates hypertension due to reduction of blood vessel lumen. Similarly excessive generation of ROS, particularly H_2_O_2_, has been detected in cancer cells. Though the exact sources of H_2_O_2_ generation in cancer cells are not known, the higher amount of H_2_O_2_ increases level of hydroxyl radical (OH), which in turn mediates oxidative damage of DNA and ultimately results in genomic instability. Transcriptional activation of some genes like cyclooxygenase-2 (COX-2), matrix metalloproteinases (MMPs), and cyclin B1 has been reported to be due to oxidative stress or induced generation of H_2_O_2_ [21–23]. Overexpression of these genes and H_2_O_2_ generations are also observed in breast cancer tissue, colon cancer, and cervical cancer tissue. Compelling evidence from epidemiological, clinical, and experimental studies demonstrated that phenolic and other naturally occurring compounds present in cereals, legumes, nuts, olive oil, vegetables, fruits, tea, and red wine exert antioxidative/anti-inflammatory effect through their free radical scavenging properties, as well as enhancement of antioxidative enzymes [18, 20].

Phytochemicals like curcumin, resveratrol, terpenoids, epigallocatechin-3-gallate (EGCG), and isothiocyanates share common properties and play an important role to activate the phase II detoxifying and antioxidant enzymes like HO-1, GSH-Px, and glutathione-S-transferase (GST) by targeting the common transcription factor Nrf2 [nuclear factor (erythroid derived 2) related factor] [20, 24]. Epidemiological studies reported regular consumption of lycopene (rich in tomato, spinach, etc.) significantly induced the antioxidant enzymes like SOD, GSH-Px, and glutathione reductase (GR) and reduced form of glutathione (GSH); moreover this bioactive compound also reduced the levels of lipid peroxide malondialdehyde (MDA), LDL oxidation, and ROS mediated DNA damage. Additionally, lycopene was found to induce cardioprotective effect by reducing MDA levels and increasing GSH levels in postmenopausal women [25, 26]. The redox-sensitive transcription factor Nrf2 plays an important role in regulating induction of phase II detoxifying or antioxidant enzymes (HO-1, SOD, etc.) which result in cellular defense against oxidative stress and exert cytoprotective mechanism [27]. Many researchers have elucidated the molecular mechanisms responsible for activation of Nrf2. A cytoskeleton binding protein called Kelch-like erythroid CNC homologue- (ECH-) associated protein 1 (Keap1) binds to Nrf2 regulating its translocation to the nucleus or its activation [28]. Following nuclear translocation, Nrf2 binds not only to the specific consensus* cis*-element called antioxidative responsive element (ARE) or electrophile response element (EpRE) present in the promoter region of genes encoding many antioxidant enzymes but also to other* trans*-acting factors such as small Maf-F/G/K as well as the coactivators of ARE including cAMP response element binding protein (CREB-binding protein or CBP), p300 that can coordinately regulate the ARE-driven antioxidant gene transcription [28–30]. Tea extract contain large amount of polyphenols (theaflavins, catechins, epicatechins, epicatechins-3-gallate, EGCG), which are characterized by 2 or more aromatic rings with at least one hydroxyl group linked by a carbon bridge. Hydroxyl group of tea polyphenols (TPs) are actually the main source for electron donor and efficiently scavenging singlet oxygen (e.g., NO, peroxynitrite).* In vitro* study by Leung et al. found significant reduction of oxidized LDL following application of theaflavins and catechins [31]. Similar response was also observed by Lee et al. showing decrease in oxidized LDL in plasma of individuals after 4 weeks of green tea consumption [32, 33]. Many researchers have reported that antioxidative properties of tea polyphenols (theaflavins and catechins) might be due to inhibition of ROS-generating enzymes (e.g., iNOS), which contribute for the production of NO mediated free radicals. The molecular mechanism of antioxidant enzyme induction by EGCG or other TPs and polyphones still remain unexplored in large extent [28]. A widely accepted model for induction of ARE-mediated antioxidant gene expression involves phosphorylation of serine/threonine residues of Nrf2 by protein kinases, leading to enhanced nuclear accumulation of Nrf2 and subsequent ARE binding. Furthermore, Wu et al. observed induction of HO-1 enzyme in endothelial cells due to activation of Akt and Nrf2 by EGCG, which impart protective measure of endothelial cell against H_2_O_2_ mediated oxidative stress [34]. Supplementation of curcumin as well as EGCG has been reported to enhance Nrf2 nuclear translocation and upregulation of HO-1 by Akt, EKR1/2, and p38 MAPK signaling in human breast epithelial cells as wells as B lymphoblasts [27, 28, 35]. Other plausible mechanisms of EGCG-induced Nrf2 activation are oxidation or modification of cysteine thiols present in Keap1 by ROS and/or via active form of EGCG during its redox-cycling [28]. Although EGCH mediated ARE-mediated upregulation of antioxidative gene may also be plausible through activation of MAPKs, which finally activate Nrf2. However, in contrast many studies have reported that inhibition of MAPKs by phytochemicals finally induced Nrf2 activity [27, 28]. Procyanidin B2 was found to increase nuclear translocation of Nrf2 via regulation of ERKs and p38 signaling [36]. Similarly soy isoflavones and polyphenols rich in red wine, tea, and dark chocolate regulate vascular reactivity by targeting endothelial nitric oxide synthase (eNOS) and inducing nuclear accumulation of Nrf2. Induced endothelium-dependent NO generation in response to phytochemicals (polyphenol) may further activate cellular sensor(s) for oxidative stress and thereby enhance NO bioavailability [37]. NO can react further with superoxide anions (O∙^−^_2_) to form peroxynitrite, which further upregulates nuclear accumulation of Nrf2 and activates ARE dependent transcription of phase II and antioxidant defense enzymes [37, 38]. Epidemiological studies have demonstrated that supplementation of soy isoflavones for longer period improves arterial compliance in men and postmenopausal women, induces plasma nitrite/nitrate levels, and decreases plasma endothelin-1 levels. However, in contrast, an isoflavone deficient diet fed from conception throughout adult life might result in decreased GSH concentrations and mRNA levels for eNOS.* In vivo* studies on aged male rats by Mahn et al. have shown that supplementation of soy protein diet rich in genistein and daidzein, found to interact with estrogen receptors, increases mRNA levels of eNOS and antioxidant enzymes [39]. Hence, vascular protection and antioxidative effect of soy isoflavone diets mostly related to an upregulation of eNOS expression and activity, NO bioavailability in association with accumulation of Nrf2, and ARE dependent activation of antioxidant defense enzymes [37, 40]. Impaired metabolism and overproduction of ROS also contribute for neuronal degeneration and onset of neuronal diseases such as Alzheimer's disease (AD) and Parkinson's disease (PD). Therefore, neutralization of ROS and other types of free radicals by endogenous antioxidative enzymes (HO, GST) or regulation of oxidative stress by inducing supplementary intake of natural antioxidant is considered as a primary preventive therapeutic measure for the clinicians to protect from chronic neuronal disorder. Numerous pieces of evidence reported curcumin (rhizome of* Curcuma longa*), EGCG (tea), and resveratrol (grapes, berry) as potential natural occurring bioactive neuroprotective compounds because of their antioxidative properties [41, 42]. Curcumin has been shown to exert protective effect against neuronal degeneration by scavenging ROS and neutralizing NO induced free radicals.* In vivo* study on Alzheimer's disease (AD) transgenic mouse model by Lim et al. demonstrated that curcumin reduces neuronal oxidative stress by inducing expression of cytoprotective proteins or antioxidant enzymes such as superoxide dismutase (SOD), catalase (CAT), glutathione reductase (GR), glutathione peroxidase (GPx), heme oxygenase 1 (HO-1), and glutathione-S-transferase (GST) [43]. Additionally curcumin is also able to increase the intracellular glutathione (GSH) pool by affecting the nuclear content or by triggering specific transcription factors such as 12-tetradecanoate 13-acetate (TPA), electrophilic response element (EpRE) [44, 45]. Reversal of intracellular GSH pool in AD patient by curcumin makes it unique therapeutics for AD treatment as depletion of cellular GSH level plays a pivotal role in AD pathogenesis. However because of curcumin's low water solubility and poor bioavailability the major challenge to use curcumin as therapeutic agent for the treatment of AD is to cross the blood brain barrier (BBB) [42]. However EGCG, main phenolic component of green tea, has been reported to have slow rate of BBB penetration and about 5% bioavailability following oral consumption [46]. Series of rodent studies have shown the pleiotropic effect of EGCG, which exert the neuroprotective measure by modulation of antioxidative enzyme activity (SOD, GST) in association with suppression of ROS generation and protection of neuronal cells from glycation induced neurotoxicity.

Resveratrol is one of the most widely distributed phenolic compounds in fruits (apple, berry, and grapes), nuts, and so forth. Extensive study over resveratrol has shown its antioxidant properties and protective role in many chronic health issues like neural disorder (AD, PD), inflammation, CVD, and so forth. Central nervous system is one of the target organs for resveratrol as it is able to cross the BBB, although bioavailability of resveratrol is very low as it is metabolized quickly to glucuronide and sulfate conjugate. Similar to curcumin and EGCG, administration of resveratrol also mediates its neuroprotective effect via stimulation/upregulation of various antioxidant enzymatic activities such as HO, GST, and SOD. Additionally administration of resveratrol on transgenic mouse model of AD has shown protective role of this particular phytochemical against neuronal impairments mainly through inhibition of NF-*κ*B modulated expression of several pathways like iNOS, prostaglandin E2(PGE2) [47–50]. Recent study by D'evoli et al. has shown the antioxidative and cytoprotective effects of red chicory leaf extract to improve intestinal complications are mainly due to its high content of both anthocyanins and phenolic compounds. Molecular targets of chemopreventive dietary phytochemicals are nuclear transcription factors NF-*κ*B and hypoxia inducible transcription factor (HIF) [51]. This finding has been further supported recently by Bak et al., who demonstrated that wild grape seed procyanidins (WGP) effectively suppressed the generation of oxidative stress mediators like ROS and nitric oxide (NO) mainly by preventing the activation of NF-*κ*B and p38 mitogen-activated protein kinases (MAPKs) pathway in LPS-RAW264.7 cells [52]. Bak et al. have also shown that antioxidative and chemopreventive effects of WGP are associated with induction of nuclear factor E2-related factor 2 (Nrf2)/antioxidant response element (ARE) pathway in the human hepatoma HepG2 cell line [52].

Induced oxidative stress and oxidative modification of LDL by ROS are one of the key risk factors for atherosclerotic plaque formation, which restricts blood flow and results in high blood pressure. Numerous phenolic/flavonoid compounds are potent inhibitors of LDL oxidation and exert their cardioprotective role by inducing antiplatelet and anti-inflammatory effects at localized (microvascular) and/or systemic level. Additionally polyphenols may also increase HDL levels and improve endothelial function. The mechanism of antioxidant activity of phenolics/flavonoid compounds can be characterized by direct scavenging or quenching of oxygen free radicals, which are mostly attributed to the o-dihydroxyl group in the A and/or B ring (catechol group) of their diphenylpropane structure [53]. Catechol type flavonoids (e.g., quercetin, heliosin) therefore possess powerful antioxidant activity.

Human study by Aviram et al. reported small but significant (6%) decrease of lipid peroxidation in plasma following 2 wk consumption of pomegranate juice (PJ, 50 mL/day) by 13 healthy, nonsmoking men aged 20–35 years compared with plasma obtained before study entry [54]. Additionally, a significant (9%) increase in plasma total antioxidant status was also observed after 2 wk of PJ consumption. Supplementation with 50 mL PJ/d for additional one week resulted in a further 21% decrease in plasma lipid peroxidation, whereas an additional increase in PJ supplementation to 80 mL PJ/d for another week did not inhibit plasma susceptibility to lipid peroxidation further. The inhibitory effect of PJ consumption on plasma lipid peroxidation was maintained for 2 wk after PJ supplementation ended. This study therefore showed that daily consumption of PJ may reduce the progression of atherosclerotic lesions by reducing the plasma lipid peroxidation and by virtue of its ability to attenuate platelet activations [54].

## 4. Role of Phytochemical in Inflammatory Process

Inflammation is defined as a series of immunological, biochemical, and/or cellular alterations in response to exogenous or endogenous stimuli. Both chronic and acute phase inflammatory processes act locally and systematically to activate the cells associated with inflammatory process (macrophages, endothelial cells, and fibroblast) to induce the inflammatory mediators like ROS, NO, prostaglandin E2, and proinflammatory mediators such as cytokines, TNF-alpha, and COX-2. Multiple studies have shown that overexpression of proinflammatory genes including TNF-alpha and interleukin is associated with activation of transcription factor NF-*κ*B [55]. Activated transcription factor translocates to the nucleus and either regulates the release of inflammatory mediators or induces the upregulation of inflammatory gene expression by binding with the DNA. Furthermore, phosphorylation of MAPK plays important role in chronic inflammation by regulating production of NO and proinflammatory genes from macrophages as well as in the activation of NF-*κ*B [52, 56]. Hence, suppression or inhibition of those inflammatory/proinflammatory mediators is one of the major targets for treatment of many chronic diseases (cancer, CVD, and diabetes) using anti-inflammatory compounds.

Wild grapes procyanidins (WGP) induced anti-inflammatory effect and the molecular mechanism of procyanidins was studied in detail by Bak et al. using lipopolysaccharide (LPS) stimulated RAW 264.7 [52]. In this study Bak et al. have shown that incubation of RAW 264.7 cells by WGP significantly blocked inflammatory phenomenon by reducing protein expression of iNOS and COX-2, two important inducible enzymes which play critical role in NO, PGE2 generation [52]. Moreover, authors have shown that WGP treatment significantly reduced LPS stimulated expression of proinflammatory cytokines such as tumor necrosis factor *α* (TNF-*α*) and interleukin-1*β* (IL-1*β*) in RAW 264.7 cells. These effects of WGP might be due to suppression of nuclear factor-*κ*B (NF-*κ*B) activity via downregulation of MAPK and p38 pathways. Hence, suppression of NF-*κ*B by WGP would be an important protective measure to reduce the chances of inflammation mediated chronic health issues, as transcription factor NF-*κ*B activity plays a central role in inflammatory process [52]. Similarly brassica derived phytochemicals like sulforaphane (SFN), phenethyl-isothiocyanate (PEITC), and indole-3-carbinol (I3C) are also found to exert anti-inflammatory effect by downregulation of LPS induced expression of COX-2, iNOS in mouse macrophages mainly by inhibiting NF-*κ*B pathways [57–59].* In vivo* study on C57BL/6 showed that pretreatment of mice with SFN resulted in significant reduction of dextran-sodium-sulfate induce colitis compared to PBS treated control mice [60]. Similar to brassica phytochemicals, tea polyphenols, primarily EGCG, epicatechin-3-gallate (ECG), and epigallocatechin (EGC) work as chemopreventive compounds by inducing anti-inflammatory effect in cancer cells. Both EGCG and theaflavins reduced LPS-induced TNF-alpha generation and also iNOS expression by preventing activation of NF-*κ*B [19, 61].* In vivo* study by Chen et al. demonstrated that tea flower extract (TEE), rich with many polyphenols (EGCG, EGC, and ETC), possesses anti-inflammatory effect against chronic inflammation. In this study authors have shown that oral administration of TEE in mice is associated with significant reduction of tissue specific (liver) acute inflammation by blocking cytokines (TNF-alpha, IL-1*β*) expression and NO production [62]. Recently increasing interest on beneficial effect of extra-virgin olive oil (EVOO) is mainly focused on the anti-inflammatory effect of phenolic compounds present in the EVOO. Glycoside oleuropein, hydroxytyrosol, and tyrosol are the major phenolic component of EVOO and have been able to inhibit inflammation by blocking eicosanoids (prostaglandin I2, Leukotriene B_4_) production enzymes such as COX-2, lipoxygenase (LOX), and phospholipase A2 (PLA2) in animal and human cells [63]. Additionally, hydroxytyrosol one of the main component of EVOV reported to impart anti-coagulatory/anti-atherosclerotic effect in association with their anti-inflammatory activity in individuals with type 1 diabetic by reducing the production and accumulation of thromboxan B2 (TXB2) and hydroxyeicosatetraenoic 27 acids (HETE) in serum, which results in reduced platelet aggregation. Moreover, this particular phenolic component of EVOV can also be considered as potent anti-inflammatory agent as this is able to prevent the expression of COX-2 and iNOS in LPS-stimulated macrophages [64, 65].

Polyphenol present in red wine and black tea, for instance, quercetin, EGCG, ECG, and theflavins, are able to inhibit COX-2 and LOX in dose dependent manner following application to LPS activated murine macrophage RAW 264 cells [66]. Likewise, kaempferol, a flavonoid widely distributed in many natural sources including apples, grapes, cabbage, and tomato, significantly reduces inflammation by inhibiting the generation of PGE2. Researchers have shown that cocoa polyphenols (flavonols, anthocyanidins, catechins, etc.) decrease the inflammation by numerous mechanisms such as inhibition of mitogen induced activation of T cells and reduced expression of IL-2 and other cytokines (IL-6, TNF-alpha). Curcumin has been identified as naturally occurring active component with wide range of medicinal effects against many chronic diseases like CVD, cancer, and metabolic disorder due to its strong antioxidative and anti-inflammatory response* in vitro* and* in vivo*. Aggarwal and Harikumar reported oral application of 70–100 mg/kg curcumin reduces systemic (plasma) and tissue specific (aortic tissue) inflammatory response as well as LDL oxidation and hypocholesteromic effects in rodents [67]. Similarly, Nemmar et al. have shown that supplementation of curcumin as oral gavage (45 mg/kg) in male mice significantly reduces systemic inflammation by preventing the release of TNF-*α* and C-reactive protein (CRP) [68]. CRP is an acute phase protein and has been identified to play an important pathogenic role in the progression of many chronic degenerative diseases like CVD, arthritis. Increased level of CRP has been observed in almost every inflammation mediated diseases onset. Similar to curcumin Pauwels et al. have shown antiarthritic/inflammatory potentials of type-A procyanidine polyphenol (TAPP) extracted from cinnamon bark. Rheumatoid arthritis (RA) is an acute inflammatory condition in joint with functional impairments mainly involved with localized (skeletal join) excessive prostaglandin synthesis as well as systemic inflammation characterized by increased level of serum CRP. Oral consumption of cinnamon bark extract and its polyphenol (procyanidine) has been found to regulate systemic inflammation by reducing CRP and was also able to stimulate autoimmune system by blocking expression of many inflammatory mediators (prostaglandin E2, NO) in RA rats [69]. Results showed the therapeutic value of this particular polyphenol and its potential to reduce or reversal of RA progression. Due to its strong anti-inflammatory potentials TAPP is found to be successfully used to regulate airway hyperresponsiveness (AHR) or asthma by reducing inflammatory mediators like IL-4 and IL-13, which played critical role in mucus hyper-secretion and the onset of AHR [69]. Recently Hazewindus et al. have shown synergistic effect of bioactive compounds such as lycopene, ascorbic acid, and *α*-tocopherol, rich in tomato. In this study authors have shown that lycopene alone significantly exerts anti-inflammatory effect by reducing the release of proinflammatory cytokine TNF-*α* via regulation of NF-*κ*B activation. Moreover significant inhibition of lipid peroxidation by the combination of ascorbic acid and *α*-tocopherol is the complementary to the anti-inflammatory effect of lycopene.

## 5. Role of Phytochemicals in Metabolism Regulation

Metabolism is the cascade of cellular chemical transformation regulated by a pool of enzymes which break down the organic matter, harvest the energy (digestion), and allow the cells to grow, reproduce, and respond to molecular signal by maintaining inter- and intracellular homeostasis. Any alteration of this chain of chemical event may lead to metabolic disorder or unregulated metabolism which ultimately results in high plasma glucose level (diabetic), obesity, high blood pressure (hypertension), CVD, organ failure, and so forth. Diseases or cluster of physiological changes induced mostly by metabolic disorder like diabetes, CVD are the major health concern for morbidity with high socioeconomic burden worldwide. Modification of dietary pattern in association with regulation in life style (regular exercise, weight loss) plays an effective role to slow the deleterious effect metabolic syndrome [70]. Epidemiological, randomized, and controlled dietary studies on human or rodents provide a lot of evidence showing that consumption of dietary fibers improves the indices of diabetes risk by regulating glycemic and plasma glucose level. The important pathogenic factors responsible for the development of metabolic disorder, insulin resistance, *β*-cells dysfunction, and finally diabetes are oxidative stress and tissue specific (localized) and/or systemic inflammation. Phytochemicals present in the whole grains including phytosterol, flavanols, anthocyanidins, and cinnamic acids modulate the diabetic risk by exerting their antioxidative and anti-inflammatory effect [70, 71]. Increasing number of controlled epidemiological studies has reported significant reduction of plasma cytokines (IL-6) or CRP in healthy individuals following regular consumption of rye bran and whole wheat bread. Furthermore, short-chain fatty acid of cereal fibre prevents inflammatory response in colonic mucosa by binding to the G-protein coupled receptor and also by blocking transcription factor NF-*κ*B. Similarly consumption of brown and black rice for 4 months was found to exert cardioprotective effect by regulating oxidative stress in individuals with preexisting complications, coronary heart disease (CHD) [72]. Although the detailed molecular mechanism is still unexplored, controlled epidemiological studies have reported the significant reduction of oxidized plasma malondialdehyde and urine prostaglandin in subjects with CHD. Similar to wholegrain, fenugreek seed and cinnamon extract has been widely used for its medicinal value in the treatment of diabetes. The hypoglycemic and hypocholesterolemic effect of fenugreek seed is mainly attributed to its high concentration of soluble fiber content, which helps to decrease the postprandial blood glucose [70]. TAPP, the main active component of cinnamon extract significantly lowered the blood HbA1c level and also improved the insulin signaling in murine models of diabetes [73]. Hypoglycemic effect of blueberry extract in humans is mainly due to presence of anthocyanin and myrtillin. Significant reduction of blood pressure in salt-sensitive spontaneously hypertensive rats (SHRs) supplemented with 3% blueberry enriched diet for 8 weeks might be due to vasodilator effect of blueberry polyphenol through an endothelium mediated stimulation of NO metabolism and activation of COX-induced product [74, 75]. Similarly, grape seed extract (GSE), which is a rich source of polyphenols (approximately 90% of which are procyanidins and 7% other polyphenol compounds), has become popular for the treatment and prevention of chronic cardiac disease and other disorders.* In vivo studies on *spontaneously hypertensive rats have shown that regular consumption of 0.5% GSE-supplemented diet significantly reduced the arterial blood pressure possible due to induction of endothelium dependent vascular dialtion [76]. Aldolase reductase (AR), a rate limiting enzyme in polyol pathways plays an important role in the progression of diabetic complications and other chronic metabolic disorder. Detailed phytochemicals analysis by Termentzi et al. has shown* Sorbus domestica* fruits extract content high concentration of flavonoids and hydroxycinnamoyl esters. Further evaluation on* in vitro* study of* Sorbus domestica* fruits extract indicates high content hydroxycinnamoyl esters possess AR blocking activity. Thus regular consumption of this particular fruit extract can be the promising natural therapeutic measure for regulation of chronic or long-term diabetic complications [77, 78]. Furthermore, Russian tarragon (*Artemisia dracunculus* L.) extract was demonstrated to attribute antidiabetic/antihyperglycemic effect in both streptozotocin induced genetically diabetic KKAy murine models.* In vitro* study on hepatic cells showed that bioactive components like 6-demethoxycapillarisin and 2′,4′-dihydroxy-4-methoxydihydrochalcone are involved in hepatic glucose output by inhibiting the transcription factor for a primary enzyme, phosphoenol pyruvate carboxykinase (PEPCK) [79]. Regular consumption of fresh bitter melon juice (popular vegetables of Asian origin) or dried whole vegetables have been found to significantly regulate the blood glucose level and diabetes-related complications including nephropathy, insulin resistance, and early cataract formation. The observed hypoglycemic effect of bitter melon might be due to presence of cucurbitane-type triterpenoids steroidal saponins called charantins, insulin-like peptides and alkaloids, which are associated with hypoglycemic activity [70]. Similar as bitter melon, antidiabetic effect of garlic was not well documented in human studies, but garlic was found to be an effective in regulating blood glucose level in streptozotocin-induced as well as alloxan-induced diabetes mellitus in rats and mice. Although the exact mechanism of garlic as an antidiabetic agent is not clearly understood, but several* in vitro* studies proposed that allicin (main component of garlic) may enhance serum insulin following effective combination with cystein, or garlic can simply exert its antidiabetic effect by inducing secretion of insulin from the pancreatic beta cells or its release from bound insulin [80, 81]. However, cardioprotective role of garlic has been well documented both* in vivo* and controlled human studies mainly by regulation of metabolism and due to its anti-atherosclerotic effect. Many* in vivo* studies reported supplementation of 1–4% garlic and garlic protein diet in hypercholesterolemic rats significantly reduced serum cholesterol, triglyceride, and LDL cholesterol. Similarly, controlled human studies have shown that a dose of 4 grams of garlic cloves per day, 6 days a week for 6 months, significantly affects the LDL-C or other plasma lipid concentrations in adults (30–65 years) with moderate hypercholesterolemia [82]. Garlic extract contains high levels of water soluble phytochemicals such as S-allyl-cystein (SAC) as well as small amount of oil soluble compounds [14].* In vitro* studies further demonstrated that this cholesterol-lowering effect of garlic might be because garlic triggers the formation of allicin through action of alliinase enzymes and allicin inhibits cholesterol synthesis* in vitro* [83, 84]. Even* in vitro* studies reveled that antiatherosclerotic and antiatherogenic activities of garlic are due to reduction of lipid content and/or LDL oxidations by allicin and SAC present in the garlic extract [80]. Moreover, antihypertensive effect of garlic extract was also evaluated both in hypertensive rats and in controlled human (hypertensive) studies. The gamma-glutamylcysteine component of garlic might be responsible for lowering blood pressure by inhibiting angiotensin-converting enzyme* in vitro* [14, 80, 85]. Similar to organosulfur compound in garlic, phytosterols (including sitosterol, campesterol, and stanols) are also efficiently inducing cholesterol lowering effect both in animal and in human studies. The primary sources of phytosterols are vegetables, nuts, fruits seeds, and vegetable oil (olive oil, canola oil). Sitosterol and campesterol are the most frequent plant sterols and constitute about 60% and 35%, respectively, of plant sterols in food [86]. Phytosterols and cholesterol are structurally similar but are metabolized differently. Due to their structural similarity to cholesterol, plant sterols are well studied for their ability to inhibit cholesterol absorption. In addition to their cholesterol lowering effect, plant sterols may possess antiatherosclerosis, anti-inflammation, and antioxidative activities [87]. Pelletier et al. demonstrated that consumption of 0.7 g of soy sterols fed to 12 normocholesterolemic individuals reduced LDL cholesterol by 15.2% relative to the control [88]. Many* in vitro* studies on caco-2 cells have shown that the hypocholesterolemic effect of plant stanols might be by reduced intestinal cholesterol due to structural similarity between plant stanols and cholesterol [89].

Phenolic compounds, namely, chlorogenic acid and ferulic acid, and a plant alkaloid, berberine, are also considered as potent antidiabetic agent with high therapeutic efficiency as well as less side effect. All these three phytochemicals are reported to enhance the uptake of 2 deoxyglucose (2DG) in time- and dose-dependent manner. Reduced glucose transporter (GLUT4) translocation along with impaired glucose transport is the major pathogenesis of diabetes progression. Treatment of L6 myoblasts with these above mentioned phytochemicals shows that chlorogenic acid and berberine significantly upregulate the expression of GLUT4 and peroxisomal proliferator-activated receptors-gamma (PPAR-*γ*) expression whereas treatment of L6 myoblasts with ferulic acid results in significant induction of GLUT4 and phosphoinositide-3-kinase (PI3K) gene expression. Based on this study researchers concluded that chlorogenic acid and berberine exert their antidiabetic effect or increased glucose uptake in PI3K independent manner; however increased glucose uptake with ferulic acid is highly dependent on PI3K pathway [90, 91]. Several* in vivo* and* in vitro* studies have demonstrated the synergistic effect of resveratrol, quercetin, and genistein in preventing obesity/weight gain and adipogenesis and also contribute to control multiple metabolic disorders like dyslipidemia, insulin resistance, and so forth. Hypolipidemic effect of resveratrol has been found to exert significant reduction of serum total cholesterol, triglycerides, and lipid content in hepatic tissue in high-fat diet hamster [92]. Lagouge et al. revealed that the protecting activity of resveratrol on regulation of metabolism is mainly achieved due to enhancement of sirtuin 1 (Sirt1), a NAD+-dependent deacetylase activity, which improving the cellular insulin sensitivity. Therefore, consumption of plant based diet imparts beneficial effect by preventing the development and progression of age chronic diseases that are highly associated with metabolic disorder [93].

## 6. Role of Phytochemicals in Cell Migration and Proliferation

Abnormal proliferation and migration of vascular smooth muscle cells (VSMC) are the primary event contributing to the pathogenesis of atherosclerosis or restenosis and is also linked with other cellular process such as localized inflammation, arterial hypertension due to narrowing of blood vessel. Similarly excessive cell proliferation and impaired programmed cell death or apoptosis represent major causative factors for the development and progression of cancer [19]. Therefore, this particular event is considered as a main target for the reversal of the neovascularization mediated health effect (atherosclerosis, cancer, etc.). Series of* in vitro* and* in vivo* studies has demonstrated tea as healthy beverage as it is rich source of many polyphenols (catechins, EGC, and EGCG) and is capable of reducing or slowing the progression of atherosclerosis by inhibiting the SMC proliferation and arrested the cells in G1 phase [94, 95]. EGCG is found to be effectively inducing SMC arrest by blocking cyclin D1 and cyclin E; beside this EGCG is also inhibiting the cell growth markers PCNA [94, 95]. Tea polyphenols exert antiproliferative effects by interacting also with matrix metalloproteinase (MMP) system. Expression of various MMPs has been found to be upregulated mostly in any type of cancer cells. Many studies have already shown that MMP transcription is induced and regulated by many regulators like cytokines, growth factor, ROS, and so forth. Several MMPs have been found to be as key agonists in tumor invasion, metastasis, and angiogenesis, including MMPs 1, 2, 3, 9, and 14 [96–98]. Two particular subtypes of this family, MMP 2 and MMP 9, are found to be actively involved in the turnover of basement membrane collagen and alteration of matrix proteins during angiogenesis and also plays important role for tissue remodeling. Both catechins and EGCG reduce MMP2/MMP-9 secretion in VSMC by preventing NfKB and AP-1 activities [95]. Khan et al. have reported administration of green tea polyphenol (e.g., EGCG) in transgenic adenocarcinoma mice results in significant inhibition of cancer progression or metastasis of prostrate tissue by regulating MMP 2 and MMP 9 expression [99]. Additionally it has been shown that both catechins and EGCG suppress PDGF-BB induced activation of PDGF-receptor mediated signal transduction pathways in VSMC by blocking tyrosine phosphorylation and their downstream molecular targets [99]. These findings are consistent with our recent study on antiproliferative effect of* Gentiana lutea* and its main constituent isovitexin where Kesavan et al. have reported aqueous root extract of* G. lutea* and isovitexin significantly inhibit PDGF-BB induced proliferation of SMC by downregulation of ERK1/2 and iNOS expression [100]. Similarly in another study we have shown significant antiproliferative effect of ellagic acid (EA), phenolic compound present mainly in barriers, which might be due to prevention of PDGF-BB receptor tyrosine phosphorylation/activation, reduced ROS generation, and downstream stimulation of ERK1/2 [101]. In this study authors have also demonstrated that treatment of streptozotocin-induced diabetic rats with EA reduced the onset of atheroma formation by blocking SMC proliferation and downregulation of cyclin D1 expression [101]. Hence all these findings suggest that regular consumption of phytochemicals is associated with prevention of cardiovascular complications. Antiproliferative effect of phytochemicals are not only inhabit the onset of CVD progressionbut also plays a significant role in cancer prevention. Curcumin and its derivatives have been found to inhibit the proliferation of breast cancer (BC) cell lines and by downregulation of matrix metalloproteinase-1 (MMP-1) expression [102]. Resveratrol is also effectively preventing cell proliferation by stimulating cell cycle arrest via regulation of cell cycle proteins such as cyclins E and D1. Furthermore, resveratrol induces apoptosis by controlling series of events including upregulation of tumor suppressor genes p21Cip1/WAF1, p53, the proapoptotic protein Bax expression, activating caspase apoptotic signals, and downregulation of antiapoptotic proteins Bcl-2, Bcl-XL, and survivin expression. Likewise, quercetin may also act as a plant derived anticancer drug by upregulating expression of Bax, which finally leads to cell apoptosis and is able to impart antiproliferative effect by suppression of Bcl-2 protein activity and stimulation of DNA fragmentation procedure [103, 104]. Many studies reported that antimetastatic effects of phytochemicals are mainly due to alteration of activity or expression of cell adhesion molecules (CD44), which are significantly upregulated during metastasis and primarily contribute to the cancer cell growth. Ouhtit et al. have shown that treatment of BC cell line with cocktail of six phytochemicals (indole-3-carbinol, resveratrol, genistein and curcumin, C-phycocyanin, quercetin) caused a marked inhibition of proliferation and motility of cells in combination with suppression of cell adhesion molecule CD44 expression, which plays an important role as a metastasis initiating factor [102].

## 7. Role of Phytochemicals in Angiogenesis

Angiogenesis is a complex controlled phenomenon for growth and development with proangiogenic and antiangiogenenic factors. Angiogenesis is well orchestrated physiological balance between the stimulatory and inhibitory signals for new blood vessels development or preexisting vasculature. Any alteration in normal angiogenesis results in either poor vascularisation or abnormal vasculature. Chronic ischemic wound is the result of insufficient blood vessel formation or reduced angiogenesis. In contrast cancer cells or tumors induced uncontrolled angiogenesis or abnormal blood vessel growth to spread metastasis by secreting proangiogenic factors like vascular endothelial growth factors (VEGF). In a very recent work Kowshik et al. have demonstrated antiangiogenic effects of ellagic acid in a hamster model of oral oncogenesis by examining the transcript and protein expression of hypoxia-induced VEGF signaling cascade. In this study authors have found ellagic acid significantly inhibiting angiogenesis in hamster buccal punch maybe by abolishing phosphatidylinositol-4,5-bisphosphate 3-kinase (PI3K/Akt) and MAPK and VEGF signaling pathways, which involves suppression of histone deacetylase 6 (HDAC6) and hypoxia-inducible factor 1-alpha (HIF-1*α*) responses [105]. Wound healing capacity of* Tephrosia purpurea* was studied by Lodhi et al. in rats with three different types of wound such as incision wound, excision wound, and dead space wound. Histopathological study following treatment of wound area with* T. purpurea* ethanolic extract has shown significant increase in angiogenesis or blood vessels formation, fibroblast cells, and collagen fibres generation due to presence of large amount of flavonoids in the extract [106]. Several* in vivo* and* in vitro* studies have shown combination of EGCG and curcumin exhibited synergistic growth inhibition of premalignant and malignant cells through the suppression of angiogenesis, cell proliferation, and upregulation of apoptosis [107]. Similarly Kowshik et al. have reported role of astaxanthin as a cancer preventive agent by suppression of angiogenesis. Astaxanthin, which is a non-provitamin A carotenoid predominantly distributed in microalgae, fungi, plants, and sea foods, is found to inhibit the onset of tumor progression by targeting signal transducer and activator of transcription 3 (STAT3)/Janus kinase 2 (JAK-2) [108].

## 8. Summary: Future of Phytochemicals and Therapeutic Mechanism

**Figure 1:**
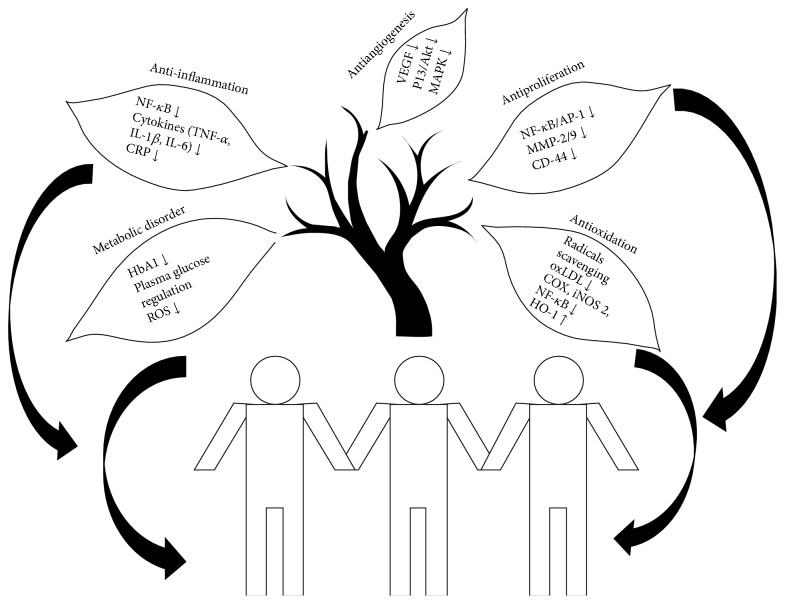
The molecular mechanism/signaling targeted by phytochemicals to exert the protective effect: antioxidation, anti-inflammation, antiproliferation, metabolic disorder, and antiangiogenesis.

Relationship between phytochemicals and disease prevention has been a major focus of health research for almost half a century. Epidemiological and clinical studies indicate that the risk of chronic or noncommunicable diseases is reduced by a diet rich in fruits, vegetables, and unrefined grains. Other foods such as mono- and polyunsaturated fats, brans, nuts, plant sterols, and soy proteins have all been shown to have a favorable effect on pathogenesis of CVD (e.g., lipid profile and blood pressure lowering effect), cancer, and/or neurodegeneration. The progression in the knowledge of both the disease pathomechanisms and the targeted pathways by dietary components to exert their medicinal effect may provide new avenues to develop dietary strategies to prevent and/or to treat the numerous disorders. Based on the epidemiological and/or clinical evidence, it has been found that phytochemicals and/or naturally occurring active compounds are having broad range of physiological effects, which include reduction of inflammatory cascades, oxidative stress, improved metabolic disorder, vascular homeostasis, or antiproliferation (Figure 1). However, it is still not clear whether an individual component of the diet or a combination of nutrients and dietary habits is responsible for the observed protective effects. Therefore, screening of large scale of potential beneficial molecules present in the regular diet may provide lead molecules that may be used in the future as inexpensive dietary supplements specific to disease prevention. The products being naturally occurring in the markets would be easily available for all strata of the society. This would also open up a huge possibility of herbal product based markets and scopes of employment. The area of phytochemicals and its protective effect will only grow successfully if preclinical and/or clinical research is able to integrate credible science with thorough consumer understanding, uncompromised taste, and convenience, along with awareness about the preventive role of dietary product on the development of chronic diseases.

## Figures and Tables

**Table 1:** Classification of phytochemicals (adapted from Gonzalez-Castejon and Rodriguez-Casado [36, 92, 107]).

Dietary phytochemicals	Functional derivatives		Sources	Therapeutics effect
Polyphenols	Phenolic acid Curcuminoids Flavonoids Chalcones Stilbenes Lignans Isoflavonoids	Caffeic acid Ferulic acid Chlorogenic acid Curcumin Flavonols, Flavanones Anthocyanins Flavanonoles Phlorizin Resveratol Sesamin Pinoresinol Genistein Daidzein	Coffee beans, soybeans Turmeric Tea, fruits (citrus, apple, grapes) Vegetables Tomatoes, tea Grapes, red wine Whole grains, legumes Soya	Antioxidative effect: (1) Curcumin, resveratrol, tea polyphenol, and isothiocyanates **↑ **the phase II detoxifying and antioxidant enzymes like HO-1, GST by modulation transcription factor Nrf2 [20, 24]. (2) Curcumin protects against neuronal degeneration by ↓ ROS neutralizing NO induced free radicals. Curcumin ↑ intracellular GSH pool by triggering specific transcription factors (e.g., TPA, EpRE) [44, 45]. (3) Isoflavones target eNOS and redox sensitive gene expression and regulate vascular reactivity [37, 40]. (4) Aged rats fed soy protein diet rich in genistein and daidzein, which interact with estrogen receptors, ↑ eNOS and antioxidant enzymes expression [39]. (5) Resveratrol, anthocyanin, blocking activation of NF-kB, MAPK, and PGE2, protect neuronal disorder [51, 52]. Anti-inflammatory effect: (1) Procyanidins ↓ expression of iNOS and COX-2 inhibit inflammatory phenomenon. Procyanidins also significantly ↓ expression of TNF-*α* and IL-1*β* by blocking NF-kB activity via ↓ of MAPK and P38 pathways [52]. (2) Tea and/or red wine polyphenol inhibit expression of COX-2, LOX and exert anti-inflammatory effect. Similarly kaempferol, a flavonoid rich in fruits, vegetables (broccoli) ↓ reduce inflammation by inhibiting the generation of PGE2 [64–66]. (3) Oral supplementation of curcumin (45 mg/kg) in male mice ↓ stemic inflammation by blocking the release of TNF-*α* and CRP [68]. (4) Cinnamon bark extract (content TAPP) protects from AHR or asthma by reducing inflammatory mediators like IL-4 and IL-13 [69]. (5) Cinnamon bark extract protects against systemic inflammation in rheumatoid arthritis by reducing CRP and stimulating autoimmune system by ↓ expression of many inflammatory mediators (prostaglandin E2, NO) in RA rats [69]. Metabolism regulation: (1) Anthocyanin and myrtillin of blueberry extract show hypoglycemic effect in humans. Supplementation of 3% blueberry enriched diet for 8 weeks and/or 0.5% GSE-supplemented diet significantly reduced the arterial blood pressure in SHRs via endothelium mediated stimulation of NO metabolism and activation of COX-induced product [74, 75]. (2) TAPP, the main active component of Cinnamon extract, significantly ↓ the blood HbA1c level and improves the insulin signaling in diabetic animal study [73]. (3) Phenolic compounds, namely, chlorogenic acid and ferulic acid, and a plant alkaloid, berberine, are considered as potent antidiabetic agent, as these phytochemicals enhance the uptake of 2DG in time- and dose-dependent manner and significantly upregulate the expression of GLUT4 and PPAR-*γ* and PK13K expression [90, 91]. Antiproliferative effect: (1) Tea polyphenols exert antiproliferative effects by interacting with MMP system. Tea polyphenol ↓ SMC proliferation by blocking cyclin D1 and Cyclin E and/by inhibiting the cell growth markers PCNA [94, 95]. (2) Resveratrol ↑ apoptosis by upregulation of tumor suppressor genes p21Cip1/WAF1, p53, the proapoptotic protein Bax expression, ↑ caspase apoptotic signals, and ↓ antiapoptotic proteins Bcl-2, Bcl-XL, and survivin expression [103, 104]. (3) Polyphenols (resveratrol, genistein, curcumin, C-phycocyanin, and quercetin) inhibit proliferation and motility of cells by suppression of cell adhesion molecule CD44 expression [102]. (4) Ellagic acid, isovitexin ↓ SMC proliferation might be by ↓ ROS generation and ↓ of ERK1/2 and iNOS expression [101]. Antiangiogenic effect: (1) Ellagic acid significantly ↓ angiogenesis in hamster buccal punch by ↓ PI3K/Akt and MAPK and VEGF signaling pathways, suppressing HDAC6 and hypoxia-inducible HIF-1*α* responses [105]. (2) *Tephrocia purpurea* rich in flavonoids was found to exert wound healing effect by significant ↑ of angiogenesis or blood vessels formation, fibroblast cells, and generation of collagen fibres [106]. (3) Astaxanthin (non-provitamin A carotenoid) predominantly distributed in microalgae, fungi, plants, and sea foods, inhibits tumor progression by regulating STAT3/JAK-2 [108].

Terpenoids	Carotenoids Sesquiterpenes	Lycopene Lutein Carotene Acyclic compound (Farnesol, Nerolidol) Cyclic compound (Abscisic acid)	Tomatoes, spinach, carrot Fruits, vegetables	Antioxidative effect: consumption of lycopene (rich in tomato, spinach, etc.) significantly ↑ antioxidant enzymes SOD, GSH-Px, GR, and GSH and ↓ levels of MDA in hypertensive patients. Lycopene ↓ MDA levels and ↑ GSH levels in postmenopausal women and protects from cardiovascular disorder. Anti-inflammatory effect: lycopene ↓ the release of proinflammatory cytokine TNF-*α* by ↓ NF-kB activation and induces anti-inflammatory effect. Moreover significant inhibition of lipid peroxidation by the combination of ascorbic acid and *α*-tocopherol is the complementary to the anti-inflammatory effect of lycopene.

Organosulfur		Allicin Allyl sulfide	Garlic, onion	Metabolism regulation: (1) *In vivo* antidiabetic effect of garlic is well documented in diabetic rats and mice. Allicin (main active component) induces pancreatic secretion of insulin or its release from bound insulin [80, 81]. (2) Garlic and garlic protein diet significantly ↓ serum cholesterol, triglyceride, and LDL cholesterol by allicin mediated inhibition of cholesterol synthesis [83–85]. (3) Gamma-glutamylcysteines component of garlic ↓ blood pressure by inhibiting angiotensin-converting enzyme [14, 80, 85].

Phytosterols	Sterol, Stanols, Campestanols	Diosgenin	Fenugreek, wild yam	Metabolic regulation: phytosterol, inhibiting cholesterol absorption [83, 85, 89]
